# Mothers' perspectives on virtually delivered online video feedback parenting interventions: a qualitative study

**DOI:** 10.3389/fpsyg.2026.1633270

**Published:** 2026-05-05

**Authors:** Tarendeep K. Johal, Anja Wittkowski, Ming Wai Wan

**Affiliations:** 1 Division of Psychology and Mental Health, School of Health Sciences, The University of Manchester, Manchester, United Kingdom; 2 Greater Manchester Mental Health NHS Foundation Trust, Manchester, United Kingdom; 3 Manchester Academic Health Centre, The University of Manchester, Manchester, United Kingdom; 4 Perinatal Mental Health and Parenting Research Unit, Greater Manchester Mental Health NHS Foundation Trust, Manchester, United Kingdom

**Keywords:** maternal perspectives, mother-infant, parenting programs, remote delivery, video-aided

## Abstract

**Introduction:**

While video feedback (VF) parenting programs are well-evidenced for improving maternal sensitivity and mental health, how well they translate to remote virtual (online) delivery has been little researched. The aim of this qualitative study was to understand mothers' perspectives on taking part in a hypothetical virtually delivered VF parenting intervention.

**Methods:**

Women with a child under 24 months of age living in the UK were interviewed to provide their views on the potential benefits and barriers if they were to participate in a VF parenting program. Data were analysed using thematic analysis.

**Results:**

Ten mothers took part and were interviewed. Thematic analysis yielded five main themes: 1) Accessibility is enhanced but barriers to access remain, 2) Video is a powerful yet exposing tool, 3) The online digital nature of the intervention creates uncertainties, 4) The healthcare professional's qualities and the process of building trust, and 5) Understanding the intervention's requirements and integration into family life. Although virtual VF interventions offer increased accessibility and convenience, mothers also emphasized the need for additional scaffolding and tailored support to mitigate technical, practical, and psychological challenges that they perceived to be associated with remote delivery.

**Discussion:**

Given that the virtual format demands additional emotional and practical resources—particularly for parents who may benefit most from parenting support—future interventions should incorporate parents' perspectives, including those of non-participants and non-completers, into their design process to optimize engagement and effectiveness.

## 1 Introduction

Early relational difficulties between an infant and their primary caregiver can have lasting impacts on the infant's emotional and social development, contributing to the intergenerational transmission of mental health difficulties (Fonagy et al., 2023; Girme et al., 2021). Attachment theory has been particularly influential in highlighting the importance of parent-infant relationships for the infant's psychological development (Bowlby, 1969; Fonagy et al., 2023). Parenting interventions were developed to support this early relationship and enhance infant outcomes. In practice, they are often delivered within infant and perinatal mental health teams, social work and family support services, and early education settings, where they typically target caregivers experiencing mental health difficulties, elevated stress, or broader contextual risks. In these contexts, video feedback (VF) interventions have emerged as a distinctive and well-evidenced approach that uses filmed parent–infant interactions as a basis for guided reflection. While specific models vary in how video material is used and how feedback is delivered, VF interventions typically share two core components: the parent reviews previously recorded video footage of their interactions with their infant alongside a trained practitioner, and these observations form the basis of guided discussions, which are often—in a structured program—focused, reflective and center on a particular theme. The VF technique is often the central organizing principle of an intervention program; however, VF can also be employed as one component of a multi-technique approach.

The main purpose of VF-based programs is usually to increase caregiver sensitivity toward their infant, aligning them closely to attachment-based approaches (Bakermans-Kranenburg et al., 2003; Fukkink, 2008; Green et al., 2008; O'Hara et al., 2019). Although VF models vary in their theoretical underpinnings—ranging from attachment-focused approaches, such as VIPP (Video-feedback Intervention to promote Positive Parenting; Juffer et al., 2008), to strengths-based relational models like VIG (Video Interaction Guidance; Kennedy et al., 2010) and Marte Meo (Vik and Rohde, 2014), to more directive, coaching-oriented programs based on behavioral principles—most share a common aim of enhancing the parent's capacity to notice, interpret, and respond to their infant's cues, usually across multiple sessions. In attachment-based frameworks, parental interactive behaviors are theorized to reflect their internal working model of attachment, which in turn shapes the infant's developing expectations of trust and relationships with others. Many VF programs also target broader caregiver outcomes, such as enhancing perceived parenting competence, self-confidence, and wellbeing, and reducing stress (Butler et al., 2020; Fukkink, 2008). For parents of toddlers and older children, some programs focus on reducing behavioral difficulties (e.g., de Oliveira et al., 2024; Juffer et al., 2017), drawing on social learning principles and more directive practitioner guidance (e.g., Ousley et al., 2022; Butler et al., 2020).

A recent qualitative meta-synthesis highlights that across these diverse models, parents experience the direct observation of their own interactions—paired with a supportive, non-judgmental stance from the practitioner—as particularly powerful and transformative (Wan et al., 2025). VF is thought to work through mechanisms that slow down interactions and draw attention to subtle cues, enabling parents to notice behaviors that are easily missed in real time and to develop greater reflective functioning and insight into their child's intentions. The practitioner's strengths-based or positive approach helps reduce defensiveness, build confidence, and create the emotional safety needed for deeper reflection. Guided discussion about short, carefully selected clips supports parents to link observed behaviors to underlying needs and to consolidate adaptive interactional patterns. These active ingredients—attuned practitioner support, emotional containment, fine-grained observation of interactional moments, and the evocative experience of seeing oneself on screen—are considered central to VF's effectiveness.

VF interventions have been well-evaluated, with evidence supporting benefits for parenting behavior and child outcomes (Bakermans-Kranenburg et al., 2003; Balldin et al., 2018; Fukkink, 2008; O'Hara et al., 2019). Bakermans-Kranenburg's et al. (2003) large-scale meta-analysis of 70 studies found in their subgroup analysis that attachment-focused interventions that incorporated VF (12 studies) were more effective in improving observed relationship outcomes than those without. Extending beyond VF programs that are attachment-based, O'Hara et al.'s (2019) meta-analysis of 20 randomized controlled trials (RCTs) and quasi RCTs found moderate evidence for improved parental sensitivity following VF, although the effects were comparable with other similar interventions.

The COVID-19 pandemic of 2020–2021 accelerated developments in virtually delivered parenting interventions, largely due to a sudden shift from the traditional in-person format to a virtual online format almost overnight to keep studies going while adhering to national lockdown rules, although their effectiveness had been little evaluated (Solís-Cordero et al., 2022). Virtual delivery of parenting programs were perceived by parents as acceptable and feasible (Van Ijzendoorn et al., 2023) and the format has subsequently been valued by practitioners for its flexiblity and destigmatising approach (Papakonstantinou Rodi et al., 2024; Philipp et al., 2023). Some practitioners have expressed that rapport was potentially stronger online relative to in-person contact due to having the parent's full attention on the screen (Van Ijzendoorn et al., 2023), while others found establishing rapport online more challenging (Papakonstantinou Rodi et al., 2024). Yet this shift in delivery occurred during an unprecedented event. Some parents might have felt grateful for any support at all (Loose et al., 2023; Russell et al., 2020). Persistent barriers such as limited access to suitable devices, reliable internet, or a confidential space at home also continue to shape who can meaningfully engage with virtual provision.

Since the end of the pandemic, remote parenting interventions have become standard practice, yet little is known about parents' post-pandemic perspectives. Virtual interventions have been shown to effectively support parents of older children with behavioral difficulties (Baker et al., 2015; Feil et al., 2020; Hicks and Baggerly, 2017; Linhares et al., 2022; Sadeghi et al., 2021). However, VF interventions differ from other parenting approaches in their reliance on video technology and on the parent's coordination of attention between the practitioner and the screen. In infancy-focused programs, this format introduces distinctive challenges: capturing naturalistic parent-infant interactions, editing footage, and facilitating parental reflection on subtle cues may all be compromised by virtual delivery. Close observation of subtle parent-infant interactions may be challenging in a virtual format (Hippman et al., 2025), particularly when using a mobile phone as their primary device (Callicott et al., 2021) and video recordings may fail to fully capture non-verbal communication (Blair et al., 2024).

Virtual delivery places additional demands on parents, often requiring technical and organizational skills to record, upload, and submit videos for co-reviewed feedback discussions. Compared with in-person sessions, virtual interactions may feel less fluid, be more vulnerable to technical disruptions, and lack some of the embodied cues that support therapeutic connection. These challenges are amplified in VF, in which the quality of the footage directly shapes what can be observed, discussed, and reflected upon. Environmental distractions such as childcare responsibilities, the presence of family members, or background noise can further interrupt the flow of sessions, and unlike in home-based visits, practitioners have limited ability to help parents manage the setting in real time. A post-pandemic pilot study offering mothers with perinatal mental health difficulties a choice of virtual or blended VF delivery found that most preferred in-person, home-based sessions (Barnicot et al., 2024), suggesting that while virtual formats increase accessibility, they may not always provide the relational containment or practical support or ease that parents value in infancy-focused VF work.

VF parenting programs are well-established, evidence-based interventions, and remote delivery has increasingly become part of routine practice. However, the VF technique's reliance on technology, shared parent-practitioners observations, and nuanced, practitioner-guided reflection may pose challenges for virtual delivery. This small qualitative study aimed to understand the views of mothers with a child under 24 months of age and living in the UK with respect to participating in a hypothetical virtually delivered VF-based parenting intervention (if they were offered the service). While previous studies obtained views of parents who had participated in an intervention (excluding those who would take part), this study focused on understanding mothers' views of a virtually delivered VF parenting intervention if they were to be offered to participate in one.

## 2 Method

### 2.1 Study design

This study adopted a qualitative, exploratory design to examine mothers' views on the hypothetical acceptability of a virtually delivered VF-based parenting intervention. Conducting individual semi-structured interviews enabled mothers to describe their expectations, perceived benefits, and concerns in their own words, and to explore how they felt about engaging with VF in a virtual format. A convenience sampling strategy was used to recruit mothers of infants under 24 months living in the UK, ensuring that the perspectives captured were relevant to those most likely to be offered such an intervention. Interviews were conducted online or telephone, audio-recorded, and transcribed verbatim. Ethical approval was obtained from [The University of Manchester Research Ethics] (ID Reference: 2022-13093-25665), and the study adhered to the Consolidated Criteria for Reporting Qualitative Research (COREQ; Tong et al., 2007) to support transparency, reflexivity, and methodological integrity.

### 2.2 Participant inclusion and exclusion criteria

Mothers over 18 years of age, living in the UK, with a child aged 0–24 months, were eligible for the current study. Further inclusion criteria were being the biological mother, having sufficient English fluency to be interviewed, and having videoconferencing tools or a telephone. Participants could but need not have had previous experience of taking part in any type of parenting intervention. This study did not include fathers because we chose to focus our efforts on understanding the views of the parent who was more often involved in parenting interventions, due to resource constraints. The child age range was selected based on when VF interventions focused on the parent-infant relationship tend to be offered.

Participants were recruited by an advert which had been shared on a range of (national) parenting and community websites, online parenting forums and social media, local community group networks and community libraries in Manchester city center in the UK. The advert which was designed to attract a diverse representation of mothers through the use of images invited mothers to complete a short anonymous online survey on UK mothers' views about parenting, mental wellbeing and online parental guidance. The advert mentioned the option to participate in an interview, and emphasized that no prior experience of parenting programs was needed. Following informed consent, 52 mothers completed the online survey comprising the presentation of a simplified explanation of a typical VF parenting intervention accompanied by an image (line drawing) showing a parent-practitioner discussion while co-reviewing a video of parent-infant interaction. They then completed a series of multiple choice questions inviting their views and preferences of this kind of program as well as more general multiple choice questions on their perceived parenting needs and mental wellbeing.

At the end of the survey, mothers were offered the option to leave their details to be contacted to participate in an online or telephone interview. Thus, the survey's purpose had been two-fold to obtain some descriptive data from a larger sample on mothers' views about VF programs and to recruit a range of mothers to the interview, including those who had heard of parenting interventions, which might have been challenging if approached directly for interview. The quantitative findings were not reported, primarily because the small sample size precluded statistical analysis. All survey respondents who had provided consent-to-contact were approached, and ten participated in an interview. Survey respondents were (optionally) entered into a prize draw for a shopping voucher, and interview participants were given a shopping voucher. as reimbursement for their time.

### 2.3 Interview topic guide

A semi-structured interview topic guide was developed to understand the perceived barriers to and motivators of engaging mothers in a hypothetical remotely delivered VF parenting intervention. The content covered was guided by the research team's experience of parenting intervention work, collating videos of parent-infant interaction, conducting feasibility research of parenting interventions, and by an extensive literature review of qualitative studies of parental experiences of VF-based interventions (Wan et al., 2025). Additionally, a University Community Liaison Group was consulted on the accessibility of participant-facing content, which led to the inclusion of an image in the survey to accompany a brief description of VF parenting programs and other minor wording suggestions, which were implemented. However, the interview was not formally piloted.

The topic guide covered the following areas: 1) whether they have taken part in or would ever consider taking part in a VF parenting program (generally), 2) perceived gains and worries in participating in a virtually delivered program, 3) views on feasibility of participation, including any barriers and specific preferences (including being able to record the videos themselves, preferred length of feedback sessions, and emotions mothers anticipate feeling when recording videos for use) and 4) preferred supports before and during the intervention to ease any concerns.

### 2.4 Procedure

Prior to the interview, mothers had been informed that there were many different types of positive parenting programs that had been shown to enhance parenting confidence, attachment and infant social-emotional development and that among the best evidenced was a “behavioral video feedback approach,” which involves the parent reflecting on pre-recorded videos (of the parent and baby playing with each other), guided by a professional over a number of sessions. Although VF can be employed as one component of an approach involving different techniques, we centralized the description on VF only. Both the survey content that they had completed earlier and the participant information for the interview study outlined what a VF parenting program generally involved, and it was made transparent prior to participation that the mother would not be expected to have prior knowledge on the topic, nor would her involvement lead to any invitation to participate in a VF or other parenting program. The interviewer identified themselves as a trainee clinical psychologist.

Following informed consent, the main researcher (TKJ) conducted all interviews, three of which were completed online via videoconferencing software and seven via telephone and recorded. Participants were advised to find a quiet space for the interview, but the presence of others was not monitored. At the start of the interview, a brief description of a typical VF program was provided. The description explained how parents record short videos of parent-baby play, feeding, etc., and send these to a trained health professional who would use the video to guide the parent through different exercises and review the contents of the video in feedback sessions. The mothers were informed that usually this type of therapeutic work took place in a clinic or home visit. The professional supports the parent and enhances the parent-baby relationship over several sessions. Mothers were informed that the interview would be focused on how they would feel doing this type of work online, through sending the videos online themselves and then meeting the trained health professional on an online video call. Each interview lasted 30–45 min.

### 2.5 Data analysis

Anonymized interviews were transcribed verbatim by TKJ and two psychology undergraduate students (and carefully checked by TKJ). Braun and Clarke's (2006) thematic analysis was employed due to its adaptability and practical relevance that allowed us to generate themes that were both grounded in participants' accounts and directly relevant to applied practice. The analysis was conducted in a theoretically flexible and pragmatic way, without a specific theoretical framework imposed. In this study, we aimed to identify and organize patterned responses at the semantic level to address our research aim and to represent mothers' perspectives in a grounded way.

TKJ led the analysis, with regular support of emerging codes and themes from MWW and AW, which included six sequential phases, each building upon the previous as a recursive process: 1) the transcripts were read several times to aid familiarization with the data, 2) initial codes were generated using NVivo software to capture interesting features and collate data relevant to each code, 3) potential themes were developed by collating codes, 4) themes were reviewed by checking if the themes worked in relation to the coded extracts and for the entire data set, 5) themes were defined and named, through refinement of the specifics of each theme and the overall data set (and discussed with the team), and 6) a report was produced which included selection of specific compelling extract examples relating back to the research aims of the study. Once potential themes and subordinate codes were developed by TKJ, they were discussed in detail with the supervisory team in an iterative and reflective manner, and reorganized by group agreement until consensus was reached. For reporting transparency, we note that transcripts and findings were not shared with participants.

The research team deliberated on whether or not to extend the sample, given that ten survey participants had indicated a willingness to be contacted to participate in the interviews. Here, we considered carefully data saturation, sample diversity and the practical issue of time constraints. Firstly, the main researcher found that the codes assigned to the data in the later interviews (P8–10) had been represented in earlier interviews (P1–7). No new themes emerged, and existing concepts were both well-elaborated and clearly linked to one another, consistent with criteria for code and theme saturation (Morse, 2004). Secondly, while the sample was fairly diverse in some characteristics, this was not the case for participants' educational level or marital status. Although we considered recruiting additional mothers to enhance representativeness, the limited timeframe of the project precluded such adjustment.

### 2.6 Researchers' positionality

The main researcher/first author led this study as a female clinical psychology doctoral trainee, including conducting all the interviews and leading the thematic analysis. They had no prior training in VF interventions, but had received perinatal psychology training and developed clinical interview skills. Recognizing the gaps in knowledge and experience, deliberate steps were taken by them to develop a solid understanding of VF interventions and to prepare for the role of interviewer, which included gaining an understanding of the theoretical principles underpinning VF intervention approaches and the practical elements involved in different programs. TKJ also conducted a comprehensive review of qualitative studies of parents' experiences of VF interventions, which helped to deepen their understanding of parental perspectives and concerns in a range of samples (Wan et al., 2025).

Their positionality as a non-parent and a trainee clinical psychologist at the time the research was conducted was recognized as potentially shaping both the interview dynamic and participants' willingness to disclose. Mothers had no prior relationship with the interviewer and might have assumed that the interviewer would be implicitly supportive of virtually delivered VF interventions, or conversely, might have perceived them as lacking lived parenting experience. To address this, the researcher sought to build rapport through openness, humility, and transparency about their role, while maintaining a reflective diary (Nowell et al., 2017) to document assumptions, emotional responses, and shifts in perspective throughout the research process. This reflexive practice supported ongoing awareness of how their positionality could influence both the direction of interviews and the interpretation of data.

The wider research team comprised a developmental psychologist and academic (PhD) and a clinical psychologist and academic-clinician (ClinPsyD), both female and with extensive experience in perinatal mental health and parenting intervention research. This included substantial experience in qualitative research on mothers' lived experiences and perspectives as well as extensive parent-infant interaction coding experience. In addition, MWW had received some VF-based training in two established VF approaches. Their disciplinary backgrounds and professional commitments to improving perinatal care inevitably shaped their interpretive lenses, including a sensitivity to both the potential benefits and limitations of VF interventions.

The team engaged in regular reflexive discussions during supervision meetings to help ensure a balanced interpretation that reflected participant views. For example, during the development of the themes, we regularly considered how our professional commitments might influence theme development, returning to transcripts if necessary. As no researchers were engaged in delivering parenting interventions at the time this study was undertaken, this distance helped us to approach the material without vested interest in a particular model of practice. At the same time, this position might have limited the extent to which the team could draw on practical, practice-based insights during interpretation. By making these dynamics explicit and engaging in collaborative reflexivity, we sought to ensure that both positive and critical perspectives on virtual VF delivery were represented accurately and respectfully, in alignment with participants' voices.

## 3 Results

### 3.1 Participant characteristics

Ten participants agreed to take part and were interviewed. Participant characteristics are presented in Table 1. Mothers ranged from 25 to 39 years of age and were married, in a civil partnership, or living with their partner, and half of mothers identified as White British. While all were educated to degree level and half had professional/managerial occupations (*n* = 5), income varied, with just over half of the sample reporting a household income of £52,000 to £100,000 (*n* = 6). Most (*n* = 7) mothers had recently had their first child. The youngest children across participants (3 male, 7 female) were of diverse ages up to 24 months.

**Table 1:** Sample characteristics.

ID	Age group (years)	Ethnic grouping	Education	Occupational category	Household income (£)	Marital status	Number of children	Child sex^*^	Child age (months)
1	35–39	White British	HE	Professional/senior manager	52,000 to 100,000	Married/cohabit	2	F	6–12
2	35–39	Other—NS	HE	Professional/senior manager	31,000 to 51,999	Married/cohabit	1	M	12–18
3	35–39	Asian	HE	Professional/senior manager	100,000–119,999	Married/cohabit	3	M	18–24
4	25–29	White British	HE	Associate professional	18,000 to 30,999	Married/cohabit	1	M	0–6
5	30–34	White British	HE	Professional/senior manager	52,000 to 100,000	Married/cohabit	1	M	12–18
6	30–34	White European	HE	Professional/senior manager	52,000 to 100,000	Married/cohabit	1	F	12–18
7	25–29	White British	HE	Skilled manual/services	52,000 to 100,000	Married/cohabit	1	M	6–12
8	35–39	British Asian	HE	Associate professional	31,000 to 51,999	Married/cohabit	2	M	6–12
9	30–34	White British	HE	Associate professional	52,000 to 100,000	Married/cohabit	1	M	6–12
10	30–34	Black African	HE	Skilled manual/services	52,000 to 100,000	Married/cohabit	1	F	12–18

F, Female; HE, Higher education (completed); ID, Participant number; M, Male; Married/cohabit, Married, civil partnership or cohabiting with a partner; NS, Not specified. ^*^Youngest child, when mothers had more than one child.

Two participants reported having previously taken part in a VF parenting program. When asked about parenting support that they needed or wanted, all mothers identified at least one area of support, with eight participants identifying two or more areas. Most mothers wanted practical support (*n* = 8). However, mothers also wanted help enjoying spending time with their child (*n* = 6), emotional support (*n* = 5), help with confidence in their parenting skills (*n* = 4), understanding their child's communication and signals (*n* = 4) and their postnatal mental health (*n* = 3).

### 3.2 Findings

Five main themes and four subthemes emerged from the interviews, outlined in Table 2.

**Table 2:** Study themes.

Theme	Theme name	Subtheme
1	Accessibility is enhanced but barriers to access remain	
2	Video is a powerful yet exposing tool	
3	The online digital nature of the intervention creates uncertainties and anxieties	3.1. The skills and demands of making self-recordings 3.2. Concerns about digital data security
4	The healthcare professional's qualities and the process of building trust	
5	Understanding the intervention's requirements and integration into family life.	5.1. Intervention approach and session intensity 5.2. Concerns about the manageability of information

#### 3.2.1 Theme 1: accessibility is enhanced but barriers to access remain

Almost all mothers expressed that an online format would be a significant positive because it would reduce the need to travel, preparation needed even to leave the house, and the time burden of sessions. This view was emphasized most by working mothers and those for whom travel costs were an obstacle. Most mothers cited an increased comfort in using videoconferencing platforms and greater digital literacy since the pandemic, and how online consultation was now a norm, even in healthcare:

“*it's like the way people working now has changed so much I think by incorporating the kind of style of working into healthcare will be good because people are already used to doing it*” (Participant 10).

Alongside the benefits of the online format making it more practically manageable, participants also mentioned how the home setting made it more emotionally manageable.

Several barriers to participating were also cited when considering if mothers would participate in this kind of parenting intervention, even with virtual delivery. While an online offer could ameliorate the time outlay required, almost all mothers stated that lack of time would or might prevent participation due to their full schedules with other tasks competing for time, especially for mothers who had returned to work or who had other children to care for. Some specified that the overall intervention length and the video format presented (involving creating and submitting videos of parent-infant interaction) would take up too much time and headspace:

“*Where it's already challenging to you know to juggle like everything and then to have to find the time to do it and then do all the admin, sending, communicating*” (Participant 2).

Mothers discussed anticipating technical or internet connection (e.g., buffering) issues when co-viewing videos with a practitioner. Any internet connection issues would be frustrating, especially if they arose at a time when mothers were feeling intense emotions. Anticipating such buffering or connectivity issues affected mothers' confidence in feeling safe and emotionally held within sessions. Some recognized that other mothers might not have the high quality digital device and broadband internet for virtual delivery to run well.

Mothers commonly envisaged difficulties in trying to look after their child's needs while engaging in the intervention with the practitioner, especially if no childcare provision was available for them:

“*If I was trying to review video clips with a professional in a zoom call with my baby there wanting me to play with him or do something with him I can imagine it would be quite quite difficult… and I wouldn't be able to sort of give either one my full attention*” (Participant 9).

For those from non-majority cultural backgrounds (five of the mothers identified themselves as an ethnic/racial group other than White British), potential cultural and language barriers, which could not be overcome by virtual delivery, were emphasized. Although the interviewer was not able to elaborate on the practitioner's cultural sensitivity in the context of a hypothetical program, cultural considerations were clearly important for these participants, representing an anticipated barrier.

These mothers' recommendations included the need to receive information early regarding who would be viewing the videos and where they would be viewing them, and to feel respected for their cultural beliefs and practices—such as wearing the hijab. If a program was offered only in English, then there was a question about how the program would apply to a family who did not speak English to their child at home:

“*We don't speak English to our child you see… whether that would impact the quality of the feedback or whether that would be a problem in any sort of way, because I would be very reluctant to change my, the the way I communicate with my daughter just for the videos because that again wouldn't really be a realistic depiction of how I interact with her*” (Participant 6).

#### 3.2.2 Theme 2: video is a powerful yet exposing tool

Mothers recognized video as a valuable feature of parenting programs, with most discussing its general use and a few considering its role in virtual delivery. Using video alongside discussions appealed to mothers more than relying solely on in-person observation. While eight of the ten mothers had not previously participated in a parenting intervention, they imagined that reviewing recordings of their interactions with their infant would help them notice subtle infant cues and their own responses that might otherwise go unnoticed. Video could provide tangible evidence of their parenting skills while highlighting areas for improvement, such as broadening play activities or ways to strengthen their bond with their child.

However, many mothers also expressed concerns that video use could trigger self-doubt and lower parenting confidence. Video provided an unfiltered external perspective, making self-reflection intense:

“*It's far more exposing, seeing yourself do something rather than somebody come and see you do it and then talk about it afterwards*” (Participant 1).

Recognizing this emotional discomfort, most mothers anticipated feelings of nervousness, anxiety, and self-consciousness before or during the recording process. Some feared the pressure to present an idealized version of their parenting, either to avoid judgment from professionals or from themselves. To reduce personal scrutiny, mothers preferred recording interactions involving the whole family rather than focusing solely on themselves. Nevertheless, a few mothers rationalized that with time and practice, they might grow more comfortable with video recording.

Specific to the virtual delivery model, the ability to choose which videos to send (the mothers' interpretation of taking a self-recorded video) fostered a sense of agency among mothers compared to a one-off observation in the presence of the practitioner. Some mothers indicated that if they could record their own video, this might create a greater sense of control and ownership, because they would be able to delete recordings they were unhappy with, with less pressure to have to capture “what is required” straight away. This was how some mothers interpreted the brief description of taking a video of their typical interaction with their baby, which resulted in fewer concerns and negative feelings (e.g., self-consciousness and embarrassment) about this process. They reported that if they took a self-recording that did not meet the requirements or was unsatisfactory they could record a better example. One mother, who had taken part in a VF program before, expressed enthusiasm for using self-recorded videos:

“*I think that that actually is much more helpful for boosting people's confidence rather than a quick kind of two minutes you know, you were great, you were very warm throughout, like I think it's better to see actual examples of like this is exactly how you like noticed your child's cues today*” (Participant 7).

However, their perceived ability to be selective about the video submitted led some mothers to worry about providing an accurate representation of their skills, exacerbating their self-consciousness, as described above.

### 3.3 Theme 3: the online digital nature of the intervention creates uncertainties

While mothers generally found the online format a comfortable and convenient way of participating, they held reservations about the practical demands of self-recording videos of mother-infant interaction (subtheme 1) and digital data security (subtheme 2).

#### 3.3.1 Subtheme 3.1: the skills and demands of making self-recordings

Most mothers expressed generally feeling confident that they could record themselves in a range of situations with their child and their family, but also envisaged practical issues associated with the task, particularly when managing and interacting with their child at the same time:

“*It would be difficult to be trying to be looking after him at the same time as going over across the room and trying to fiddle with the camera angle and then going back to check that I was getting him in shot*” (Participant 7).

Their digital device could distract their child from the activity, and their independently mobile child could move out of the scope of the video camera. Mothers also queried practical issues with sending the videos in advance of sessions and anticipated difficulties with having enough storage on their device, the time this would take, and technical issues. Providing a point of contact for when technical issues might occur was suggested.

The provision of high quality set-up instructions was important for enhancing mothers' confidence in the task, including clear and specific guidance on how to set up the camera, and a simple, familiar way of sending videos, such as when one shares videos on their personal social media accounts. Otherwise, managing and submitting the recordings would add to the time burden and “hassle factor,” because mothers might feel the need to do multiple “practice runs” of video recording.

#### 3.3.2 Subtheme 3.2: concerns about digital data security

While this issue did not elicit extensive content, half the mothers identified online safety as a worry. They were concerned with sending video recordings safely, where and how safely they would be stored, wanting to ensure that their videos would not be used outside of the remit they had consented for, what would happen to the videos after they had been reviewed and how long they would be kept for. Some mothers suggested that having a portal, an encrypted website or platform they could use to upload would facilitate this process and enable them to feel their recordings were safe. However, views varied and some mothers expressed having inherent trust in the clinician, the consent process and safeguarding protocols for their video recordings and had no concerns about internet security.

### 3.4 Theme 4: the healthcare professional's qualities and the process of building trust

Receiving feedback from a trained healthcare professional was highly valued by all mothers given their expertise and specialist knowledge. Engaging in discussions with a practitioner was seen as providing reassurance and helping mothers build confidence in their parenting abilities. Mothers anticipated that the practitioner's knowledge of child development would be valuable; some mothers wanted indicators of their child's progress. Cultural competence and sensitivity were important considerations for some mothers, who wanted practitioners to acknowledge diverse parenting styles and family contexts. One mother, for whom English was not the primary language spoken at home, expressed a desire for reassurance that language barriers would not affect her participation.

Nearly all mothers stated that they would prefer to meet the practitioner online before the start of the intervention to help alleviate concerns about the practitioner's communication style, qualifications, and approach, and for the opportunity to ask questions and discuss concerns before committing to the program. Establishing initial rapport would reduce anxiety and help them feel more at ease in their first sessions. Knowing in advance that the practitioner would provide supportive rather than critical feedback would be reassuring.

Thus, building trust with the practitioner would be essential, because it was considered important to mothers that the practitioner could develop an understanding of their family's unique context while also feeling confident in the practitioner's expertise. Given the vulnerability inherent in participating in a parenting intervention—particularly one delivered online—mothers expressed concerns about receiving feedback that might feel critical. They worried about or feared having negative observations pointed out, whether related to existing concerns the mother had or long-standing practices that might be framed as needing change. However, a few mothers reported that pre-intervention meetings were unnecessary, because they did not require this step to establish rapport.

### 3.5 Theme 5: understanding the intervention's requirements and integration into family life

Mothers carefully considered how the intervention would fit into their family routines, mindful of the need to balance its demands with their busy lives. A perceived net benefit was essential for engagement. Preferences varied in terms of the intervention's approach and intensity (subtheme 1) as well as the management of content and information (subtheme 2).

#### 3.5.1 Subtheme 5.1: intervention approach and session intensity

Mothers said that they would value a positive feedback approach, but it could be frustrating and unnecessary if not accompanied by actionable insights to improve on. They wanted to receive advice on their relationship with their child, on playing, feeding, behavior and meeting developmental milestones. Mothers wanted new things to try and for the practitioner to point out anything they were missing with constructive criticism.

Mothers expressed preferring different session lengths, with some mothers favoring shorter (e.g., 20 min) focused catch-up sessions and others more in-depth sessions lasting up to an hour. Some preferred a set time each week for sessions so that they could prepare; others favored more flexible session times to fit around their child's naps or their own work schedule. Many mothers took into account that they would require time to allow them to put their learning from each session into practice. Mothers' views on these issues did not seem to be influenced by the remote live online delivery format.

#### 3.5.2 Subtheme 5.2: concerns about the manageability of information

Nearly all mothers emphasized the importance of receiving clear advance information about the program's aims, benefits, and time commitment. They suggested that services should carefully consider the volume of text-based materials provided beforehand, given competing responsibilities. Some expressed concerns about the risk of information overload, advocating for concise, actionable guidance, such as small, confidence-building “nudges” that could be incorporated into future sessions. It was unclear whether this recommendation stemmed from perceived difficulties in retaining information or the anticipated emotional and cognitive demands, but it aligns with earlier themes highlighting the challenges of balancing parenting and intervention participation.

Some mothers expressed feeling uncertain about effectively capturing both themselves and their infant in self-recorded videos and five mothers requested specific, practical instructions on video recording techniques. Clear guidance on submission procedures was also considered crucial to ensure accessibility and ease of use. Most mothers preferred receiving this information via email, because it would allow easy reference and sharing with partners. Preferences for the timing of this guidance varied, with some favoring receipt one to two days before session participation, while others preferred one to two weeks in advance.

## 4 Discussion

The current study was one of the first to explore the perspectives of mothers with a child under two years of age who were asked to consider a presented hypothetical virtually delivered VF parenting intervention. Two participants had previously taken part in a parenting program, while eight had not, which might reduce the positive bias captured in previous qualitative studies that typically only included the views of parents who had chosen to engage in—and complete—this kind of intervention (Wan et al., 2025). Based on interviewing a small but culturally diverse sample of mothers, we identified five main themes that reflected parents' appreciation of and concerns about a hypothetical VF program, particularly its virtual delivery.

Mothers generally saw the appeal of a virtual VF program and felt comfortable with the proposed delivery method. However, significant barriers were also anticipated, including competing demands on their time, the need for adequate technical and childcare support, and lack of internet access for some families. Some barriers, such as cultural and language barriers and competing demands, were not ameliorated by a virtual delivery format. These concerns align with findings from previous studies on practitioner and parent perspectives regarding both virtually delivered and in-person VF interventions (Barnicot et al., 2024; Callicott et al., 2021; Labella et al., 2023; Philipp et al., 2023). Unlike prior research, however, mothers in this study had not experienced a virtually delivered VF intervention (and most had not participated in any parenting intervention), meaning their views were largely shaped by what they anticipated. This suggests that mothers form early expectations about the challenges of participating; therefore, a proactive and carefully designed approach is needed to optimize engagement.

While mothers reported that they could appreciate the use of video, they also anticipated feeling nervous, self-conscious, and fearing judgment when participating in interactive tasks that they would self-record. This finding aligns strongly with qualitative studies of parents' actual experiences of VF-based interventions (Wan et al., 2025). The mothers relayed uncertainties related to the technical nature of taking recordings to ensure they captured everything required on the screen. Interestingly, none of the mothers discussed whether or not remote participation might help to alleviate some of the nervousness and self-consciousness that mothers might feel if they participated in VF discussions. However, some liked the idea of producing their own videos of parent-infant interaction to upload, based on their interpretation of pre-session submission of recordings. While the current study proposed an approach of sending recordings in advance for feedback, others have taken a different approach of recording in-session such that instructions could be given by the practitioner at that time (Callicott et al., 2021; Van Ijzendoorn et al., 2023).

With respect to how our findings compare with past qualitative research describing the barriers to and facilitators of VF interventions experienced by parents, we note several commonalities and differences (Wan et al., 2025). Parents in the current and previous research emphasized the transformative power of video as a tool for insight, validation and change, and valued the practitioner's skill and expertise. Concerns about cultural sensitivity and cases in which English was not the language spoken at home were also raised in the current and previous works. On the other hand, parents who had participated in a program described a change in experience over time, in which they acquired a sense of ownership and empowerment, and positive changes that generalized to other relationships and areas of life (Wan et al., 2025). However, these benefits were not reported in our sample and therefore might not be anticipated by mothers. Our sample emphasized more the practical barriers to involvement (both related and unrelated to virtual delivery), whereas those who had experienced the intervention expressed strong initial anxieties about being filmed, which were reflected in our findings to a lesser degree.

### 4.1 Clinical implications

Virtual delivery offers considerable flexibility, particularly in scheduling around daily routines, and it can improve access to specialist expertise that may otherwise be unavailable. By removing geographical barriers, virtual formats enable greater reach to multilingual practitioners and culturally specialized professionals. However, successful engagement depends on addressing practical, technical, emotional, and cultural barriers that persist or that may be amplified when interventions are delivered remotely. These barriers are especially relevant not only for standalone VF programs but also for broader parenting support interventions in which VF is used as one component among others, such as psychoeducation, coaching, live modeling, or peer support.

In standalone and blended programs, VF may serve as a reflective tool to tailor practitioner feedback or reinforce learning. Yet the cognitive, emotional and logistical demands associated with self-recording (if relevant), co-reviewing footage without distractions, and interpreting subtle cues remain substantial. These demands must be anticipated and scaffolded, particularly in virtual formats. Key adaptations proposed include clear communication of goals and expectations, upfront information that is carefully presented, ensuring the intervention aligns with family routines, and reducing rigid time commitments and technical literacy demands. For disadvantaged families, integrating VF sessions into safe community spaces may be necessary.

Shorter, more frequent sessions can help build rapport with practitioners and reduce emotional intensity. Tailored support, such as advance information that can be shared with partners, flexible scheduling, and the option to discuss broader parenting concerns, can further strengthen engagement. Being in physically separate locations may act as a psychological barrier, particularly if parents feel unable to communicate emotional discomfort and disengagement. Programs should help prepare parents for what to expect at different stages of their intervention journey and offer multiple communication channels to facilitate parent-practitioner communication.

For mothers from diverse cultural backgrounds, especially those who do not primarily speak English at home, the practitioner's cultural competence and sensitivity are crucial, which should be communicated clearly and early in the engagement process. Co-designing interventions with parents, including those who might not typically engage, can help ensure that VF is embedded in a way that reflects the family's needs and values. Addressing performance pressure and ensuring positive focus during sessions are important for participation. While the task of self-recording mother-infant interactions is intended to demonstrate typical interactions, the desire to produce videos perceived as positive warrants further consideration. Some parents appreciated the sense of control and ownership that came with the idea of submitting their own videos. Further research is recommended on the impact of parents' experiences with self-selecting video recordings for intervention use.

Finally, offering an online peer support group to participants may help normalize negative feelings and provide additional support for parents. This aspect could be particularly valuable in programs where VF is one component, helping parents process their experiences and build confidence in engaging with the intervention as a whole.

### 4.2 Limitations and future research

A key limitation is the small self-selected sample, consisting only of university-educated mothers who lived with a partner. These participants were likely to have an interest in (or anxieties about) their parenting, be more reflective and articulate, and be more advantaged in having partner support. Notably, unlike previous studies that focused solely on parents who completed an intervention (Wan et al., 2025), this study captured anticipated views from mothers who had not yet participated. These perspectives—while valuable—must be understood as hypothetical and shaped by the information provided during recruitment and interview. That is, with the exception of two participants who had previously taken part in a parenting intervention (although not virtually delivered). Furthermore, almost all mothers stated in their interview that lack of time would or would likely prevent participation due to their full schedules; therefore, the sample could actually be quite typical of busy, well functioning parents and not represent those who perceive significant need for parenting support. While the study did not aim to achieve representativeness, future research should include larger and more diverse samples, including fathers who are increasingly involved in parenting programs. The requirement for participants to have access to a device and internet connection might have inadvertently excluded more marginalized groups.

This study originally utilized a mixed methods approach, but our small sample size precluded statistical analysis. A key next step is a qualitative study exploring parents' actual experiences of engaging in and completing (or not completing) a virtually delivered VF intervention, ideally as part of a pilot trial. A longitudinal qualitative approach would be particularly valuable for understanding and comparing imagined and lived experiences. While virtual formats are increasingly used in practice and research to offer flexibility, our findings suggest that several barriers must be addressed for such interventions to be optimally effective.

The interviewer's lack of practical experience in delivering VF interventions likely influenced the interviews in complex ways. On the one hand, this absence of direct practice experience reduced the likelihood of conveying an implicit bias in favor of VF approaches. The open, non-judgmental stance taken and doctoral trainee status may have also reduced demand characteristics and encouraged mothers to share authentic views. On the other hand, the lack of technical grounding might have limited the interviewer's confidence to probe or challenge specific elements of the intervention, particularly in its novel virtual format. As a result, participants tended to discuss the hypothetical program and format in a holistic way. The depth of interviews might have been constrained by the lack of probing at times, which limited the degree to which themes could be developed. A notable tendency was for the mothers to steer the conversation toward the intervention itself when redirection back toward the impact of mode of delivery (the key focus of the study) might have yielded richer insights.

Furthermore, the wider research team's previous experience with parenting interventions and parent–infant interaction research inevitably shaped the analytic lens, and while this brought depth of expertise, it might also have directed attention toward some issues over others, including our choice of themes and how they were framed. For instance, we were mindful to ensure that the practical concerns raised by mothers were given appropriate weight, recognizing our own likely tendency to focus more readily on the psychological implications of both the VF technique and its virtual delivery. With the developmental and clinical psychologists both being mothers themselves, this allowed us to give greater consideration to the barriers than might have occurred otherwise. Given the varied depth across interviews, we also reflected on how best to balance making interpretive connections between codes when participants were not always explicit, with avoiding overextending links that were only loosely suggested.

### 4.3 Conclusion

As online and virtually delivered parent-child interventions become increasingly common, this study explored how mothers with a young child viewed participating in a hypothetical virtually delivered VF-based intervention if they were offered this therapeutic approach. Mothers recognized clear advantages to this mode of delivery, particularly in terms of flexibility and feasibility for busy working mothers. However, they also anticipated the technical, practical, and emotional challenges that could undermine engagement. These concerns strongly suggest that thoughtful adaptations developed with parental input are needed to ensure that virtual VF interventions are accessible, supportive, and sustainable for families.

## Data Availability

The datasets presented in this article are not readily available because we do not have ethical approval to share raw interview data from our participants. However, reasonable requests to access other datasets should be made in writing to the corresponding author.
